# Histone Methyltransferase G9a-Promoted Progression of Hepatocellular Carcinoma Is Targeted by Liver-Specific Hsa-miR-122

**DOI:** 10.3390/cancers13102376

**Published:** 2021-05-14

**Authors:** Lan-Ting Yuan, Wei-Jiunn Lee, Yi-Chieh Yang, Bo-Rong Chen, Ching-Yao Yang, Min-Wei Chen, Ji-Qing Chen, Michael Hsiao, Ming-Hsien Chien, Kuo-Tai Hua

**Affiliations:** 1 Department of Internal Medicine, Division of Hepatology and Gastroenterology, Yuan’s General Hospital, Kaohsiung 802, Taiwan; em.yuan@parkonehealth.com; 2 School of Nursing, Fooyin University, Kaohsiung 83102, Taiwan; 3 Graduate Institute of Clinical Medicine, College of Medicine, Taipei Medical University, Taipei 11031, Taiwan; rafiyang@tmu.edu.tw; 4 Department of Medical Education and Research, Wan Fang Hospital, Taipei Medical University, Taipei 11696, Taiwan; lwj5905@gmail.com; 5 Department of Urology, School of Medicine, College of Medicine, Taipei Medical University, Taipei 11031, Taiwan; 6 Department of Medical Research, Tungs’ Taichung MetroHarbor Hospital, Taichung 433, Taiwan; 7 Department of Surgery, National Taiwan University Hospital, Taipei 100, Taiwan; yudawn@gmail.com (B.-R.C.); cyang@ntuh.gov.tw (C.-Y.Y.); 8 Graduate Institute of Toxicology, College of Medicine, National Taiwan University, Taipei 100, Taiwan; d92447001@ntu.edu.tw; 9 Department of Cancer Biology, Geisel School of Medicine at Dartmouth, Lebanon, NH 03755, USA; Ji-qing.Chen.GR@Dartmouth.edu; 10 The Genomics Research Center, Academia Sinica, Taipei 11529, Taiwan; mhsiao@gate.sinica.edu.tw; 11 Pulmonary Research Center, Wan Fang Hospital, Taipei Medical University, Taipei 11696, Taiwan; 12 Traditional Herbal Medicine Research Center, Taipei Medical University Hospital, Taipei 110301, Taiwan; 13 TMU Research Center of Cancer Translational Medicine, Taipei Medical University, Taipei 11031, Taiwan

**Keywords:** hepatocellular carcinoma, epigenetic, G9a, progression, miR-122

## Abstract

**Simple Summary:**

Targeting epigenetic alterations in hepatocellular carcinoma (HCC) provides therapeutic options in addition to traditional treatments. The aim of our study was to evaluate the potential of targeting histone methyltransferase G9a in the development of a therapeutic target. We confirmed the prognostic values of mRNA and protein levels of G9a expression in HCC respectively from public database and tissue microarray. We also confirmed the aggressive phenotypes supported by G9a in both HBV+ and HBV− HCC cells. The identification of a regulation axis between liver-specific tumor suppressor miR-122 and G9a further supported the important roles of G9a during the tumorigenesis and progression of HCC. Combination of lower miR-122 and higher G9a levels may provide prognostic potential for poor clinical outcomes and therapeutic potential for epigenetic targeting therapies.

**Abstract:**

Hepatocellular carcinoma (HCC) accounts for the majority of primary liver cancers, which is the second most lethal tumor worldwide. Epigenetic deregulation is a common trait observed in HCC. Recently, increasing evidence suggested that the G9a histone methyltransferase might be a novel regulator of HCC development. However, several HCC cell lines were recently noted to have HeLa cell contamination or to have been derived from non-hepatocellular origin, suggesting that functional validation of G9a in proper HCC models is still required. Herein, we first confirmed that higher G9a messenger RNA and protein expression levels were correlated with poor overall survival (OS) and disease-free survival (DFS) rates of HCC patients from The Cancer Genome Atlas (TCGA) dataset and our recruited HCC cohort. In an in vitro functional evaluation of HCC cells, HCC36 (hepatitis B virus-positive (HBV+) and Mahlavu (HBV−)) cells showed that G9a participated in promoting cell proliferation, colony formation, and migration/invasion abilities. Moreover, orthotopic inoculation of G9a-depleted Mahlavu cells in NOD-SCID mice also resulted in a significantly decreased tumor burden compared to the control group. Furthermore, after surveying microRNA (miRNA; miR) prediction databases, we identified the liver-specific miR-122 as a G9a-targeting miRNA. In various HCC cell lines, we observed that miR-122 expression levels tended to be inversely correlated to G9a expression levels. In clinical HCC specimens, a significant inverse correlation of miR-122 and G9a mRNA expression levels was also observed. Functionally, the colony formation and invasive ability were attenuated in miR-122-overexpressing HCC cells. HCC patients with low miR-122 and high G9a expression levels had the worst OS and DFS rates compared to others. Together, our results confirmed the importance of altered G9a expression during HCC progression and discovered that a novel liver-specific miR-122-G9a regulatory axis exists.

## 1. Introduction

Hepatocellular carcinoma (HCC), which originates from hepatocytes, is the most common type of primary liver cancer. In the past decade, HCC has not only increased in worldwide incidence [1,2], but is also a leading cause of cancer-related deaths worldwide [2,3]. HCC development is recognized as a progressive multistep process of transforming normal hepatocytes into malignant cells, primarily driven by the stepwise accumulation of genetic alterations in tumor-suppressor genes and oncogenes [4,5]. Recently, various environmental agents, such as aflatoxins and infection with hepatitis B virus (HBV) and hepatitis C virus (HCV), and lifestyle factors, such as chronic alcohol intake, that are known to be risk factors for HCC, are suspected of promoting its development by eliciting epigenetic changes [6,7,8]; however, the precise gene targets and underlying mechanisms have not been fully elucidated.

Epigenetic alterations in HCC include global genomic hypomethylation, gene-specific DNA hyper- or hypo-methylation, abnormal expressions of DNA methyltransferases (DNMTs) and histone-modifying enzymes, altered histone modification patterns, and aberrant expressions of microRNAs (miRs; miRNAs) [6,9]. Despite its significance, only limited epigenetic-based therapeutics for HCC are currently under development, and none of them have been approved for clinical use [10]. Histone methyltransferase G9a, also known as euchromatic histone methyltransferase 2 (EHMT2), catalyzes the mono- and di-methylation of histone3 lysine9 (H3K9), which are involved in heterochromatin formation, DNA methylation, and transcriptional silencing [11]. Accumulating evidence has demonstrated oncogenic roles of G9a in various cancer types, and suggested G9a as a potential therapeutic target [12,13,14,15]. High levels of H3K9 dimethylation and G9a expression were also observed in HCC [16,17,18,19]. HCC patients with higher G9a expression levels had worse survival outcomes [20,21]. Multiple functional assessments indicated that G9a may be involved in regulating proliferation, angiogenesis, epithelial–mesenchymal transition (EMT), and metastasis of HCC [19,21,22]. Regarding the above-mentioned findings supporting G9a as a critical mediator for HCC pathogenesis, inhibition of G9a methyltransferase activity with various G9a inhibitors was demonstrated to be a promising strategy for HCC treatment in preclinical evaluations [23,24]. Although recent evidence indicated that G9a is an important oncogenic driver in HCC, the mechanisms through which it regulates G9a upregulation in HCC are relatively less well-characterized. It was established that miRNAs control expressions of epigenetic regulators such as DNMTs, histone deacetylases, and histone methyltransferase, to modulate cancer progression [25,26].

Moreover, recent notifications of problematic HCC cell lines have raised concerns about previous in vitro evaluations of G9a. For example, some frequently used HCC cell lines, such as BEL7402 and SMMC7721 cells, were identified as having been contaminated by HeLa cells, and MHCC97L cells were reported to be contaminated by murine cells [27]. Another two frequently used cell lines for HCC-related studies, SK-HEP-1 and HepG2, were reported to respectively be of endothelial and hepatoblastoma origin [28,29]. It is worth noting that most of the functional evaluations of G9a in HCC were performed using these problematic cell lines [21,22,24,30]. Herein, we tried to confirm the oncogenic role of G9a in HCC progression in vitro and in vivo using several HCC cell lines which were not reported to be problematic cell lines according to the information from Cellosaurus (https://web.expasy.org/cellosaurus/, accessed on 15 December 2020) and SciCrunch (https://scicrunch.org/resources, accessed on 15 December 2020). Moreover, we discovered that the liver-specific miR-122 is a potential negative regulator of G9a in HCC cells.

## 2. Materials and Methods

### 2.1. HCC Tissue Microarray (TMA) and Immunohistochemical (IHC) Staining

A TMA containing samples from 170 patients who were diagnosed with HCC was built and none of the patients with intrahepatic cholangiocarcinoma were included in this cohort. Approval for the study was obtained from the ethics committee of Taipei Veterans General Hospital (201010021IC). Briefly, antigen retrieval of 5 µm-thick sections was performed with citrate buffer (10 mM citric acid—0.05% Tween 20, pH 6.0) and were stained with G9a by using a 1:100 dilution of anti-G9a (A8620A, R&D Systems, Perseus Proteomics, Minneapolis, MN, USA). The sections were then labeled with HRP-conjugated secondary antibody at a dilution of 1:100. Immunodetection was performed with the DAB substrate kit (vector lab, SK-4200, Burlingame, CA, USA). The sections were subsequently counterstained with hematoxylin, dehydrated, and mounted. G9a IHC staining intensities of the HCC TMA were determined via a scoring system devised of score 0 (no expression) to score 3 (highest intensity staining). IHC staining results were classified as either low or high expression according to both the intensity and extent of staining. The low-expression group was defined as having either no staining present (staining intensity score = 0) or positive staining present in ≤20% of cells (staining intensity score = 1). The high-expression group was defined as having either positive staining present in 20%~50% of cells (staining intensity score = 2) or >50% of cells (staining intensity score = 3). All of the immunostaining results were independently scored by two pathologists.

### 2.2. Analysis of Online Available Database

The CpG methylation, mRNA expression, and copy number variation (CNV) levels of G9a and the expression level of miR-122 were directly downloaded from The Cancer Genome Atlas Liver Hepatocellular Carcinoma (TCGA-LIHC) via UCSC Xena (https://xenabrowser.net/, accessed on 25 January 2020). The complete dataset was used for Normal versus Tumor analysis. Optimized cutoff values were selected and cases without survival data were excluded in the Kaplan–Meier analyses. Spearman’s rank method was used to analyze correlations between G9a mRNA expression and G9a CNV, CpG methylations, or miR-122 expression. Cases without above-mentioned data were excluded in the individual correlation analysis.

### 2.3. Cell Lines and Cell Culture

Human HCC cell lines were grown in Dulbecco’s modified Eagle’s medium (DMEM; Gibco, Waltham, MA, USA) containing 10% fetal bovine serum (FBS) (Life Technologies, Grand Island, NY, USA). All cells were grown in a humidified incubator at 37 °C and with 5% CO_2_. Huh1, Huh6, and Huh7 cells were purchased from the Japanese Collection of Research Bioresources Cell Bank (JCRB, Osaka, Japan). HepG2, Hep3B, PLC/PRF/5, and 293T cells were purchased from American Type Culture Collection (ATCC, Manassas, VA, USA). Mahlavu and HCC36 cells were obtained from Michael Hsiao at the Genomics Research Center, Academia Sinica (Taipei, Taiwan). All cells were routinely authenticated on the basis of morphologic and growth characteristics as well as by a short tandem repeat (STR) analysis and confirmed to be free of mycoplasma.

### 2.4. Western Blot Analysis

A Western blot analysis was carried out as previously described [31]. Briefly, HCC cells were harvested with radioimmunoprecipitation assay (RIPA) buffer (Merck Millipore, Billerica, MA, USA) containing a protease inhibitor cocktail (Sigma-Aldrich, St. Louis, MO, USA) and phosphatase inhibitor cocktail (Merck Millipore). Protein lysates were separated via sodium dodecylsulfate polyacrylamide gel electrophoresis (SDS-PAGE) and transferred to polyvinylidene difluoride membranes and immunoblotted with the following primary antibodies: anti-G9a (3306, Cell Signaling Technology, Danvers, MA, USA) and α-Tubulin (T5168, Sigma-Aldrich), as well as horseradish peroxidase-conjugated secondary antibodies. Blots were developed with enhanced chemiluminescence (ECL) Western blotting reagents (Merck Millipore).

### 2.5. Reverse-Transcriptase Quantitative Polymerase Chain Reaction (RT-qPCR)

Cellular messenger (m)RNA was isolated with the NucleoZOL reagent (Macherey-Nagel, Düren, Germany). Complementary (c)DNA was synthesized with a PrimerScript RT reagent kit (Takara, Shiga, Japan). The qPCR was performed according to the protocol of iTaq Universal SYBR Green Supermix (Bio-Rad, Hercules, CA, USA) using the CFX Connect Real-Time PCR System (Bio-Rad, CA, USA). Expression of miR-122 was determined with a TaqMan MicroRNA Assay (Applied Biosystems, Foster City, CA, USA) according to the manufacturer’s instructions. Fluorescence data of detected genes were normalized to the expression of actin or U6 using the 2^−ΔΔCT^ method. Primers utilized in our investigation are listed in Table S1.

### 2.6. Cell Viability and Clonogenic Assays

To detect the cell proliferation rate, HCC cells (2 × 10^4^) were seeded in 96-well plates, and their viability was assessed daily using the 3-(4,5-dimethylthiazol-2-yl)-2,5-diphenyl tetrazolium bromide (MTT) assay. Briefly, 20 μL of the MTT solution (5 mg/mL, Sigma, St. Louis, MO, USA) was added to each well and incubated for 4 h at 37 °C. After removing the supernatant, MTT-formazan crystals formed by metabolically viable cells were dissolved in 200 μL of DMSO. Finally, the absorbance was read at 595 nm. For clonogenic assays, HCC cells (500) were plated in six-well plates and allowed to grow for 10–14 days. Colonies were then fixed with 4% paraformaldehyde and stained with crystal violet (0.5% *w/v*), and colonies were manually counted using free ImageJ software (National Institutes of Health, Bethesda, MD, USA). Data were collected from three replicates.

### 2.7. Migration and Invasion Assays

HCC cells were plated in the upper chambers of uncoated (for the migration assay) or Matrigel-coated (BD Biosciences, Bedford, MA, USA) (for the invasion assay) 24-well inserts of 8 μm pore membranes in serum-free media. Cells were plated at densities of 10^5^ (Mahlavu) and 10^4^ (HCC36) cells/well for the migration assays, and 2 × 10^5^ (Mahlavu) and 2.5 × 10^4^ (HCC36) cells/well for the invasion assays. Medium supplemented with serum was used as a chemoattractant in the lower chamber. After 24 h, cells were fixed, and cells on the upper side of the filters were removed with cotton-tipped swabs. Cells on the underside of the filters were stained with crystal violet and counted under a light microscope (type 090–135.001, Leica Microsystems, Wetzlar, Germany).

### 2.8. miRNA Mimic/Inhibitor Introduction

The MirVana™ miRNA mimic and inhibitor of miR-122 were obtained from Life Technologies (Carlsbad, CA, USA) and each was transfected into HCC cells at a 10 nM concentration using the Lipofectamine^®^ RNAiMAX reagent (Thermo Fisher Scientific, Waltham, MA, USA). mirVana miRNA Mimic Negative Control #1 and mirVana miRNA Inhibitor Negative Control #1 were respectively used for each negative control. At 48 h after transfection, cells were used for G9a detection by Western blotting and functional assays.

### 2.9. Lentiviral Infection

G9a short hairpin (sh)RNAs were purchased from the National RNAi Core Facility of Academia Sinica (Taipei, Taiwan). The target sequence of G9a shRNA 1 was 5′-CAC ACA TTC CTG ACC AGA GAT-3′, that of G9a shRNA 2 was 5′-GCT CCA GGA ATT TAA CAA GAT-3′, and that of luciferase shRNA was 5′-GCG GTT GCC AAG AGG TTC CAT-3′. To construct the lentivirus miR-122 over-expressed vector (pLemirR-miR-122), a fragment encoding the pre-miR-122 sequence plus 150 bp at both 5′- and 3′-flanking regions was amplified by PCR from human genomic DNA and then cloned into the pLemirR vector (Open Biosystems, Rockford, IL, USA). Production of the shRNA lentivirus was as previously described [32]. Briefly, lentiviruses were produced by co-transfecting G9a shRNA/pLemirR-miR-122, pMD2.G, and psPAX2 constructs into 293T cells by using calcium phosphate. Viral supernatants were harvested, titered, and used to infect cells with 8 μg/mL polybrene. Puromycin at 2 μg/mL was used to select stable knockdown cells.

### 2.10. 3′-Untranslated Region (UTR) Luciferase Reporter Assay

The 3′UTR of G9a was cloned into the pMIR-REPORTTM miRNA Expression Reporter Vector (Ambion, Austin, TX, USA). 293T cells were co-transfected with the miR-122 mimic or negative control and G9a 3′UTR reporter, as well as the pRL-TK Renilla control vector (0.1 μg) (Promega, Madison, WI, USA) as an internal control for the transfection efficiency. GenMuteTM small interfering (si)RNA and DNA Transfection Reagent (SignaGen Laboratories, Ijamsville, MD, USA) were used for this transfection process according to the manufacturer’s instructions. Cells were harvested at 48 h after transfection and analyzed for luciferase activity using the Dual-Glo Luciferase Reporter Assay System (Promega).

### 2.11. In Vivo HCC Orthotopic Model

Eight-week-old male NOD/SCID (NOD.CB17-Prkdcscid) mice were housed under pathogen-free conditions and fed a diet of animal chow and water throughout the experiment. Mice randomly assigned to three groups (six mice/group) were injected orthotopically with 10^5^ luciferase-tagged Mahlavu cells stably expressing G9a shRNAs or the control shRNA. Six weeks after the injection, animals were sacrificed, and their livers were excised and examined for tumor burdens by the IVIS^®^ Spectrum in vivo imaging system (Xenogen, Alameda, CA, USA). The mouse study was performed using protocols approved by the Institutional Animal Care and Use Committee of the College of Medicine, National Taiwan University (Taipei, Taiwan).

### 2.12. Statistical Analysis

Values are presented as the mean ± standard deviation (SD). All statistical analyses were performed using the Statistical Package for Social Science software, ver. 16 (SPSS, Chicago, IL, USA). Data were analyzed using Student’s *t*-test when two groups were compared. Correlations of G9a with clinicopathologic features of HCC were examined by Pearson’s Chi-squared test. Cumulative survival was analyzed by the Kaplan–Meier method. Risk factors affecting survival were assessed by a Cox proportional hazards regression model. A *p*-value of <0.05 was considered statistically significant.

## 3. Results

### 3.1. Both G9a mRNA and Protein Levels Predict Survival Outcomes of HCC Patients

**Figure 1:**
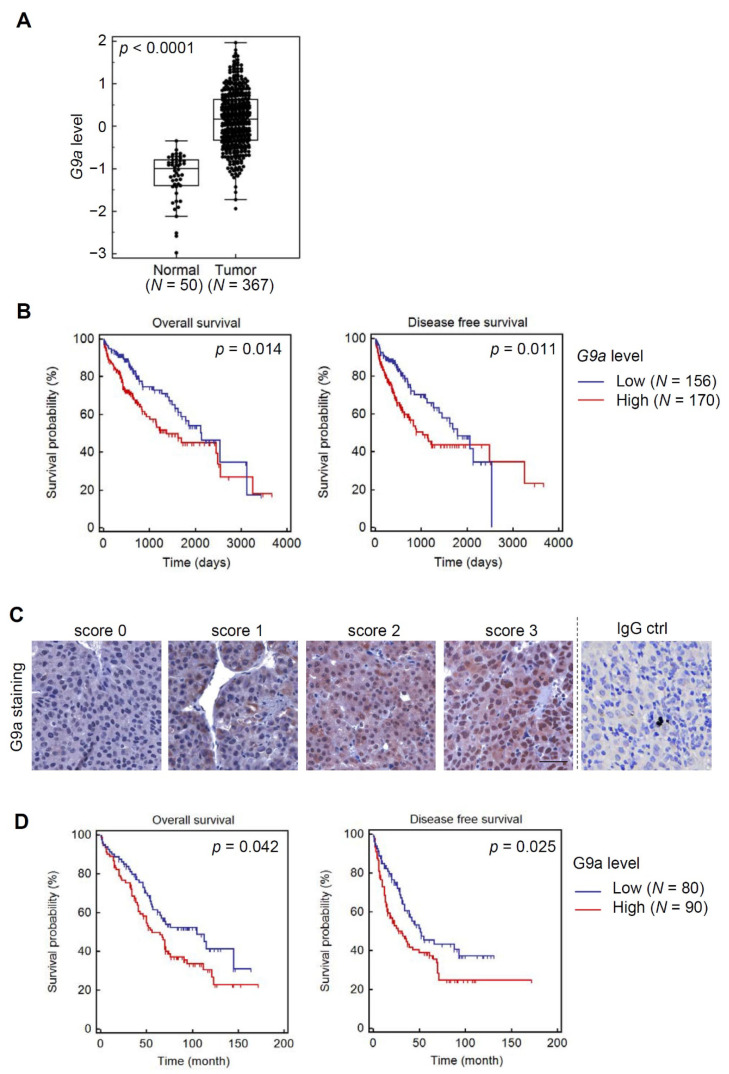
G9a mRNA and protein expression levels are prognostic markers of patients with hepatocellular carcinoma (HCC). (**A**) Box and whisker plot demonstrating a comparison of G9a expression levels between HCC and normal tissues which were downloaded from The Cancer Genome Atlas HCC dataset via UCSC Xena. This dataset shows the gene-level transcription estimates, as in log2(x + 1) transformed RSEM (reads per expectation maximization) normalized count. Unpaired two-tailed Student’s *t*-test was used for statistical analysis. (**B**) Kaplan–Meier plot of overall and disease-free survival of HCC patients stratified by G9a mRNA expression levels. A log-rank test was used to show differences between groups. (**C**) Representative photos of G9a immunohistochemical staining in HCC tissues with scores of 0–3. A negative control was also shown in which HCC tissues were incubated with normal mouse IgG replacing the anti-G9a monoclonal antibody. Scale bars, 50 μm. (**D**) Kaplan–Meier plot of overall and disease-free survival of HCC patients stratified by G9a protein expression levels. A log-rank test was used to show differences between groups.

We first compared G9a/EHMT2 levels in HCC tissues with those of normal liver tissues using TCGA database. Significantly higher G9a/EHMT2 transcripts were observed in tumors compared to normal tissues (*p* < 0.0001) (Figure 1A). Survival analysis of HCC patients in TCGA HCC dataset stratified by G9a/EHMT2 levels showed longer overall survival (OS) (Figure 1B, left panel) and disease-free survival (DFS) (Figure 1B, right panel) periods of patients harboring low G9a levels. In addition to the online database, we recruited a Taiwanese cohort with HCC and further verified TCGA results by performing IHC staining of G9a using the HCC TMA. Figure 1C shows representative examples with different G9a scores and negative staining using an IgG antibody as a control. IHC results indicated that HCC patients with higher levels of the G9a protein also had shorter OS and DFS times (Figure 1D). Relationships between G9a expression levels and clinicopathologic characteristics of HCC are summarized in Table 1. We observed that G9a protein expression levels showed a trend of correlating with cirrhosis (*p* = 0.0507), but with no other clinical characteristics in our recruited HCC cohort. Additional uni- and multi-variate analyses indicated the importance of G9a as a potential prognostic factor. The G9a score, the American Joint Committee on Cancer (AJCC) stage, and portal vein involvement were all significantly associated with OS (hazard ratio (HR) = 1.52, 1.71, and 1.74; *p* = 0.0442, 0.0148, and 0.0096, respectively). A backward stepwise multivariate analysis revealed that portal vein involvement was the only independent risk factor associated with OS (HR = 1.81, *p* = 0.0147, Table 2). We particularly noted in this analysis that the G9a score had good, although not statistically significant, prognostic potential (HR = 1.45, *p* = 0.0805). These results support the G9a expression level possibly being used as a potential predictor of a poor prognosis of patients with HCC.

### 3.2. G9a Modulates Aggressive Phenotypes of HCC Cells

**Figure 2:**
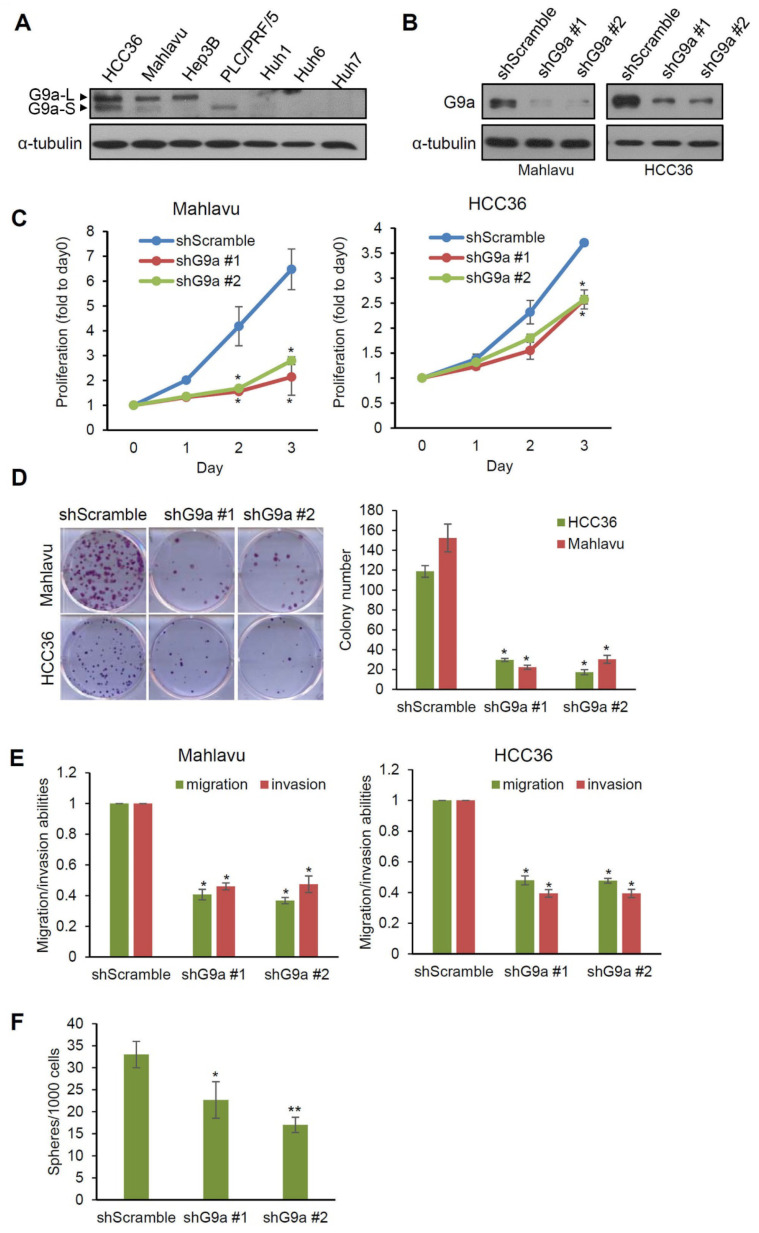
G9a is associated with aggressive phenotypes of hepatocellular carcinoma (HCC) cells. (**A**) G9a protein expression levels of seven HCC cell lines were determined by immunoblotting. The short form (G9a-S, 160 KDa) or long form (G9a-L, 180 KDa) of G9a can be detected in these cells and are indicated by arrows. (**B**) Knockdown efficiencies of two G9a-specific shRNAs were examined by immunoblotting in Mahlavu and HCC36 HCC cells. (**C**) Three-day proliferation curves were determined in Mahlavu and HCC36 cells with either G9a shRNAs or control shRNA expressions by an MTT assay. Proliferation rates were significantly lower in cells stably transfected with G9a shRNA compared to control cells. (**D**) The clonogenic cell survival assay and the quantification results of Mahlavu and HCC36 cells with control or G9a shRNA expressions. All values are the means of three independent experiments. (**E**) Transwell migration and invasion assays of G9a knockdown in Mahlavu and HCC36 cells. Quantitative plot showing multiples of the control. (**F**) Sphere-forming ability of Mahlavu cells with control or G9a shRNA expression after 2 weeks of inoculation. Three independent replicates were performed in each experiment. α-Tubulin was used as a loading control in the immunoblot experiments. * *p* < 0.05, ** *p* < 0.01. Error bars indicate the standard deviation. All uncropped immunoblotting images for this study are summarized in Figure S1.

Since functional evaluations of G9a in HCC from previous studies were mostly performed using problematic cell lines, we first selected four HBV-positive (HBV+) (HCC36, Hep3B, PLC/PRF/5, and Huh1) and three HBV-negative (HBV−) (Mahlavu, Huh6, and Huh7) HCC cell lines [33,34] which were not reported to be problematic cell lines and evaluated their G9a expression levels. As shown in Figure 2A, relatively higher G9a expression was observed in HCC36, Mahlavu, and Hep3B cells, while PLC/PRF/5, Huh1, Huh6, and Huh7 cells showed lower or undetectable levels of G9a. We next used HCC36 (HBV+) and Mahlavu (HBV−) cells for further functional analyses. G9a was knocked-down by two lentivirus-based shRNAs in Mahlavu and HCC36 cells, as verified by a Western blot analysis (Figure 2B), and then we examined the cell-invasive, migratory, proliferative, and colony-forming abilities of these HCC cells. As shown in Figure 2C, significantly lower proliferative ability of these two HCC cell lines was observed in the G9a-knockdown groups compared to their respective scrambled shRNA control cells. The effect of G9a on the long-term growth of HCC cells was evaluated by a clonogenic assay, and results showed that Mahlavu and HCC36 cells expressing G9a shRNAs grew fewer colonies than cells expressing scrambled shRNAs after 2 weeks of incubation (Figure 2D). Moreover, the effects of G9a depletion on cellular mobilities of HCC cells were also examined by transwell migration/invasion assays. Results showed significant attenuation of the migratory and invasive abilities in G9a-depleted Mahlavu and HCC36 cells (Figure 2E). Furthermore, decreasing G9a expression also suppressed the spheroid formation ability of Mahlavu cells, suggesting that G9a expression may help support cancer stem cell properties of HCC cells (Figure 2F). Collectively, the above-mentioned observations suggested that, indeed, G9a has oncogenic features in HCC cells, and also highlighted that the tumor-promoting effects of G9a may be irrelevant to the HBV status of HCC cells.

### 3.3. G9a Depletion Attenuates HCC Tumorigenicity in an Orthotopic Xenograft Model

**Figure 3:**
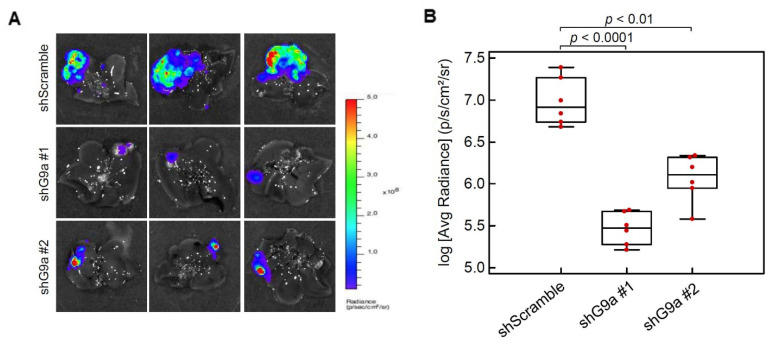
G9a promotes hepatocellular carcinoma (HCC) growth in an orthotopic mouse model. Luciferase-tagged Mahlavu cells with stable expression of control or G9a shRNA were orthotopically injected into the liver of 8-week-old NOD/SCID mice, and animals were sacrificed 6 weeks after tumor implantation. (**A**) Cancer growth of Mahlavu xenografts was imaged with bioluminescence at the end of the study. (**B**) Quantitative analysis of imaging signal intensity (photons/s/cm^2^/sr), with the mean signal for each group indicated.

Although the in vivo tumor-inhibitory potential of targeting G9a using small-molecule inhibitors or shRNAs of G9a were documented in previous studies [19,21], the in vivo models used a subcutaneous xenograft model [19] or were injected with problematic HCC cell lines such as SMMC-7211 and BEL7402 [21]. Herein, Mahlavu cells with stable luciferase expression were infected with lentiviruses carrying scrambled shRNA or G9a shRNA, and these were orthotopically injected into the liver of NOD/SCID mice. After 6 weeks of cell inoculation, we removed the liver and examined luciferase activities to assess the tumor burden. Results showed that G9a depletion obviously suppressed tumor growth compared to the scrambled shRNA group (Figure 3A). Quantification of the luciferase activities also showed remarkable reduction of the tumor burden in G9a-depleted groups (Figure 3B). These data suggest that knockdown of G9a expression in Mahlavu cells attenuated their in vivo tumor growth capacity.

### 3.4. The G9a Expression Level Correlates with Its Copy Number and DNA Methylation Status in HCC

**Figure 4:**
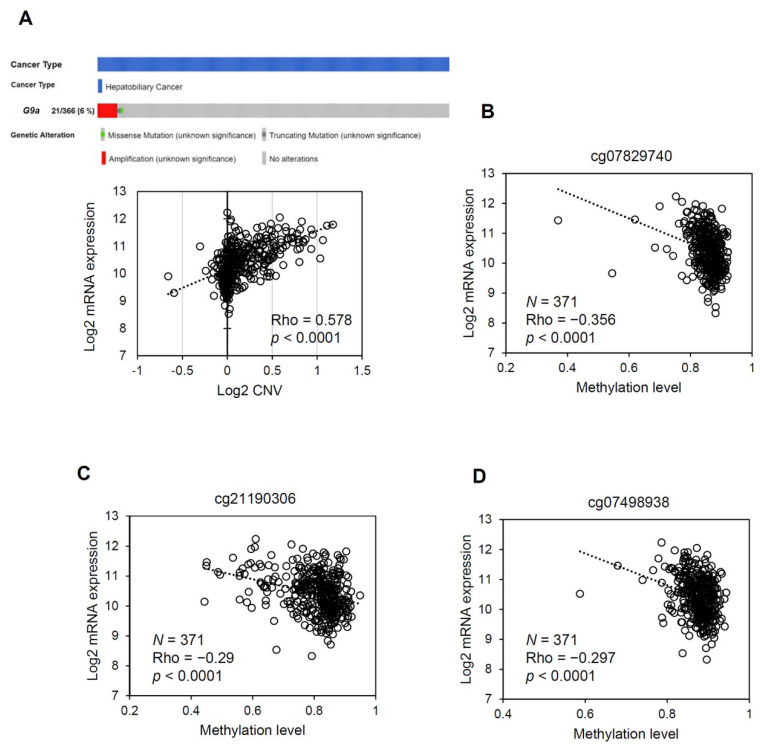
G9a expression is controlled at both the genetic and epigenetic levels. (**A**) Upper panel: OncoPrint of G9a gene alterations in TCGA HCC dataset obtained from the cBio Cancer Genomics Portal (http://cbioportal.org, accessed on 25 January 2020). Lower panel: Copy number variants (CNVs) affect *G9a* gene expression. Positive and negative values respectively indicate gain and loss of the copy number. (**B**–**D**) Correlations of indicated CpG sites and G9a mRNA expression from TCGA HCC dataset (*n* = 371). Correlations were analyzed using the Spearman rank method. Regression lines, Spearman’s rho, and *p*-values are shown on the plot. Data used for correlation analyses were obtained via the Xena platform (https://xena.ucsc.edu/cite-us/, accessed on 25 January 2020).

Until now, the underlying mechanisms that result in upregulation of G9a expression in HCC have remained largely unknown. We first examined CNVs of the *G9a* gene in TCGA HCC dataset and evaluated their correlations with G9a expression levels. As shown in Figure 4A, G9a had a frequent gain in copy numbers in HCC and had moderate (Rho = 0.578), albeit significant, correlations with G9a expression levels in HCC samples. Furthermore, only 1 sample carried a missense mutation and 1 sample carried a truncating mutation in the dataset, which indicated a low G9a mutation rate in HCC. These observations suggest that although G9a overexpression may result in part from genetic instability, G9a expression levels do not fully correspond to genetic variations. We hypothesized that epigenetic and non-coding regulators of transcription may play roles in final gene product expression levels. Therefore, we further examined the relationship between the CpG methylation status and G9a expression levels in TCGA HCC dataset. Results showed that methylation levels of some CpG sites were inversely correlated with G9a expression levels in HCC samples (Figure 4B–D, Table S2). Since the correlation between G9a expression and CNVs or the DNA methylation status only reached weak to moderate levels according to their Spearman’s Rho correlation coefficients, it can be concluded that overexpression of G9a in HCC results from multiple levels of regulation, including both genomic and epigenomic events.

### 3.5. Liver-Specific miR-122 Regulates G9a Expression, and the Growth and Motility of HCC Cells

**Figure 5:**
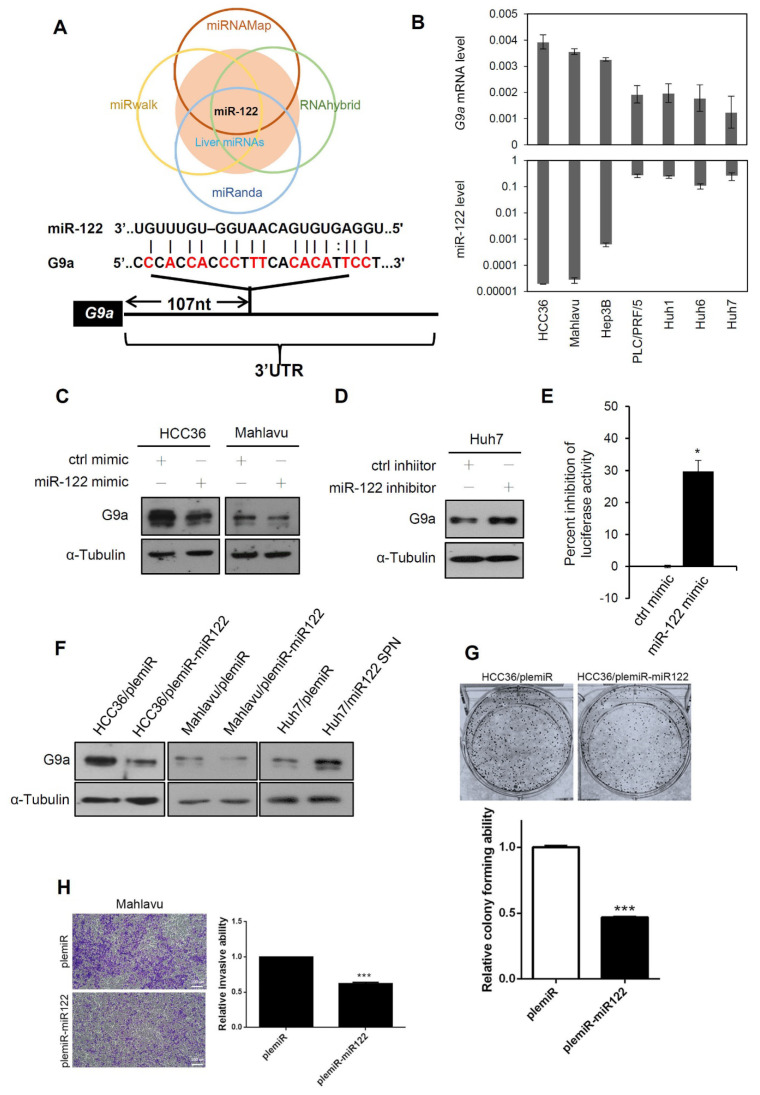
Liver-specific miR-122 regulates G9a expression, and the growth and motility of hepatocellular carcinoma (HCC) cells. (**A**) Upper panel: Venn diagram of candidate miRNAs that possibly target G9a. G9a-targeting miRNAs predicted from miRwalk, miRanda, RNAhybrid, and miRNAMap were compared and further merged with liver-enriched miRNAs. Lower panel: Predicted binding sites between the 3′-untranslated region (UTR) of G9a and miR-122 are presented. (**B**) Expression levels (2^−ΔCt^) of G9a mRNA and miR-122 were determined by an RT-qPCR. miR-122 levels are shown on a log scale. (**C**) Expression levels of G9a were determined by immunoblotting in HCC36 and Mahlavu cells transfected with a control or miR-122 mimic. (**D**) Immunoblot analysis of G9a in Huh7 cells which were transfected with the control or miR-122 inhibitor. (**E**) G9a luciferase 3′UTR reporter vector was transfected with the control mimic or miR-122 mimic into 293T cells. Luciferase activities were measured, and results are shown as the percent inhibition of the control. (**F**) HCC cells with stable ectopic expression of either miR-122 or miR-122 sponge were analyzed for G9a expression via an immunoblot analysis. (**G**,**H**) HCC cells with stable ectopic expression of either miR-122 or control vector (plemiR) were analyzed for colony-forming (**G**) and invasive (**H**) abilities. Scale bar, 100µm.Three independent replicates were performed in each experiment. Data are presented as the mean ± SD. * *p* < 0.05, *** *p* < 0.001 versus the control group. α-Tubulin was used as a loading control. All uncropped immunoblotting images for this study are summarized in Figure S1.

Mild correlations of G9a expression with CNVs and the DNA methylation status in HCC prompted us to examine other potential regulatory systems that might affect G9a expression in HCC. Altered miRNA expressions are usually observed in various liver diseases, such as liver steatosis, cirrhosis, and HCC [35,36,37]. Increasing evidence indicates that miRNAs contribute to epigenetic modulation by targeting epigenetic modifiers in various cancers, including HCC [26]. Therefore, we further used four online miRNA prediction tools (miRNAMap, RNAhybrid, miRanda, and miRwalk) to find potential miRNAs which might target G9, especially liver-specific miRNAs due to an evaluation of their liver-specific G9a regulation. To our surprise, miR-122, the most abundant miRNA in hepatocytes, was the only miRNA that fit this stringent criterion (Figure 5A). Moreover, G9a/EHMT2 and miR-122 expression levels in several HCC cell lines were analyzed, and we observed that the miR-122 expression levels tended to be inversely correlated to G9a expression levels in these HCC cell lines (Figure 5B). To evaluate the effect of miR-122 on regulating G9a expression in HCC, we transduced an miR-122 mimic into HCC36 and Mahlavu cells and examined their G9a levels. As shown in Figure 5C, G9a protein expression decreased in both HCC36 and Mahlavu cells after miR-122 mimic transfection compared to a negative control mimic. In contrast, G9a protein expression increased in Huh7 cells after miR-122 inhibitor transfection compared to the miRNA inhibitor control (Figure 5D). We further constructed a luciferase reporter containing the 3′UTR of G9a to verify the G9a-targeting ability of miR-122. Results showed that the miR-122 mimic significantly inhibited reporter activity when this vector was co-transfected with the miR-122 mimic in 293T cells (Figure 5E). Consistently, the G9a-regulatory capacity of miR-122 was also confirmed by lentiviral systems in which overexpression of miR-122 and an miR-122 sponge respectively resulted in decreased and elevated expression of G9a in HCC cells (Figure 5F). Functionally, when we transiently overexpressed miR-122 in HCC cells, the colony-forming and invasive abilities were significantly downregulated compared to control cells (Figure 5G,H). Taken together, our results indicated that liver-specific miR-122 may suppress HCC growth and motility partially through downregulating G9a.

### 3.6. Clinical Correlations and Prognostic Significance of miR-122 and G9a in HCC

**Figure 6:**
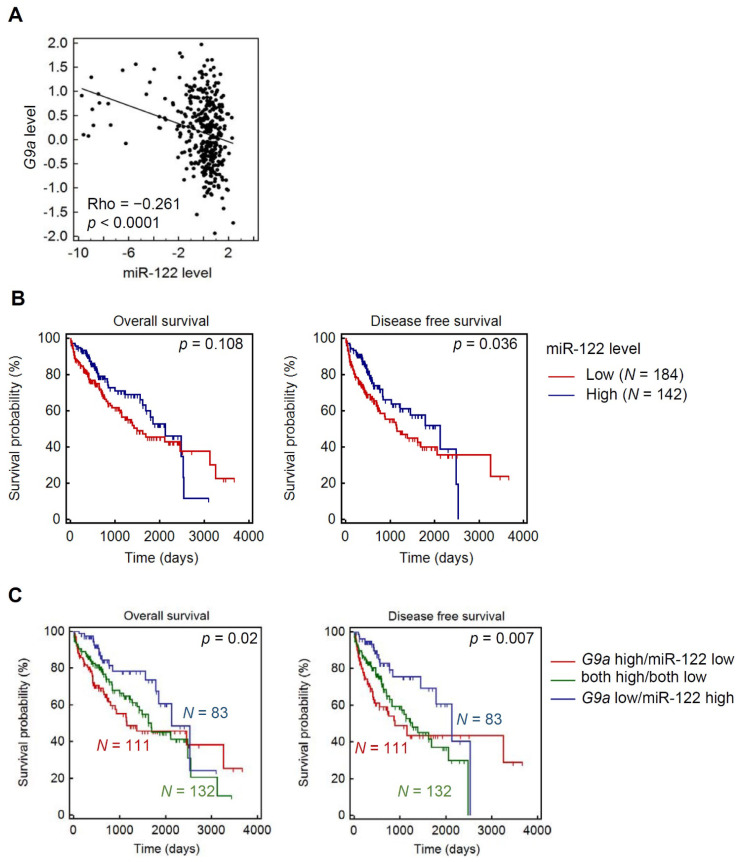
Clinical significance of miR-122 and G9a in hepatocellular carcinoma (HCC). (**A**) RNA expression scatter diagrams of G9a mRNA versus miR-122. Black dots represent expression levels of both genes from specimens in TCGA HCC dataset. Spearman’s non-parametric correlation test showing a negative correlation between G9a and miR-122 in HCC (Rho = −0.261, *p* < 0.0001). (**B**) Kaplan–Meier curves for overall survival (OS) and disease-free survival (DFS) of patients with HCC, as categorized according to high or low expression levels of miR-122. The *p*-value indicates a comparison between patients with miR-122^high^ and miR-122^low^. (**C**) All HCC patients were separated into a negative correlation of G9a and miR-122 expression, low miR-122 and high G9a, and high miR-122 and low G9a, and others (both high/both low). Data showed that patients in the G9a^high^/miR-122^low^ group had the most favorable prognosis, including both OS and DFS.

Finally, we investigated the relationship between G9a and miR-122 in clinical HCC samples using TCGA HCC dataset. A significant inverse correlation of miR-122 and G9a mRNA expression levels in HCC tissues was observed, which supports their tight regulation of HCC progression (Figure 6A). Elevated miR-122 only showed a slight trend toward favorable survival rates (*p* = 0.108 for OS and *p* = 0.036 for DFS) (Figure 6B). However, when stratifying HCC patients with both miR-122 and G9a expression levels, we observed more significant prognostic potentials in predicting both OS (*p* = 0.02) and DFS (*p* = 0.007) of patients with HCC. We found that HCC patients with G9a^low^/miR-122^high^ had the longest survival times compared to those with G9a^high^/miR-122^low^, G9a^low^/miR-122^low^, or G9a^high^/miR-122^high^ (Figure 6C). Clinical data indicated that upregulation of miR-122 and downregulation of G9a are critical events in retarding the progression of HCC and further supported our in vitro experimental findings.

## 4. Discussion

Although there are various therapeutic options available for HCC, the overall survival of HCC patients is still far from satisfactory [38]. Methylation and miRNA regulation are two key epigenetic alterations in HCC progression [39], and epigenetic modifiers have emerged as important targets for antitumor research of HCC. More than 50% of HCC cases have mutations of genes related to epigenetic regulators or chromatin-remodeling complexes [40]. Using HBV+ and HBV– HCC cell models, our study confirmed G9a as an important epigenetic regulator during the carcinogenesis and progression processes of HCC. Our results revealed that G9a expression in HCC is controlled at both the genetic and epigenetic levels. Furthermore, we first identified that miR-122 is a critical upstream regulator of G9a in HCC.

Accumulating evidence suggests that the G9a methyltransferase is a critical epigenetic regulator through catalyzing the dimethylation of histone H3K9 in both normal and pathological hepatocytes. A marked increase of H3K9me2 was reported to play a crucial role in epigenetic transcriptional gene silencing and was observed during liver maturation [41]. Liver-specific G9a-knockout (G9a-liver-KO) mice did not show significant liver injury or inflammation, but these mice had decreased cytochrome P450 enzymes (CYPs) and dysregulated lipid metabolism by hepatocytes [42]. Moreover, G9a-liver-KO mice displayed more-severe liver injury after lipopolysaccharide or acetaminophen overdose treatment [42,43]. Interestingly, other studies revealed that animals with muscle-specific G9a-knockout were resistant to high-fat diet-induced obesity and hepatic steatosis [44]. G9a expression was induced during liver fibrosis, and dual targeting of G9a and DNMT1 suppressed liver fibrogenesis in mice [45]. Liver cirrhosis is an end stage of liver fibrosis, and we actually observed that G9a expression showed a trend of correlating with cirrhosis in our recruited HCC cohort. These observations indicated that G9a was a critical mediator of liver homeogenesis and pathogenesis.

Regarding the role of G9a in liver cancer, it was reported to regulate different cellular functions of HCC, such as proliferation, migration, invasion, anchorage-independent growth, and sphere formation [19,22,24]. However, these phenomena were frequently observed in problematic cell lines [27,28,29], suggesting that further evaluations with correct cell models are required. Herein, we used two HCC cell lines of Mahlavu (RRID:CVCL_0405) and HCC36 (RRID:CVCL_VI90) to respectively represent HBV– and HBV+ HCC cells and evaluated the functional roles of G9a in both cell lines. In line with previous reports, G9a indeed participated in regulating cell proliferation, migration, invasion, and sphere-formation abilities of HCC cells. We further observed that the HBV status of these cell lines was irrelevant to the functional regulation of G9a. Consistent with these in vitro observations, clinical results of our recruited cohort and others [18] showed no correlations between G9a expression levels and the HBV or HCV status.

miR-122 is a highly abundant, hepatocyte-specific miRNA which accounts for 70% of all hepatic miRNAs in humans, and it plays a pivotal role in liver homeostasis and metabolism [46]. For example, downregulation of miR-122 is closely associated with liver diseases, such as hepatosteatosis, fibrosis, and hepatocarcinogenesis [46]. Restoration of miR-122 expression in HCC results in suppression of tumor growth and metastasis, and enhances chemosensitivity, suggesting that miR-122 functions as a tumor suppressor against HCC [47]. To date, several target genes of miR-122, such as cyclin G1, a disintegrin, and metalloprotease 10 (ADAM10), Wnt family member 1, Snail1/2, and pyruvate kinase M2, were identified to modulate hepatocarcinogenesis, the EMT, angiogenesis, and metabolism of HCC [47,48,49,50,51]. Our results indicated that G9a represents a novel, important target gene of miR-122 in regulating HCC progression. Recently, G9a was reported to be involved in DNA damage-induced liver cancer initiation. Nakatsuka et al. demonstrated that liver-specific G9a-deficient mice suppress HCC development triggered by hepatocarcinogen diethylnitrosamine (DEN). The proposed mechanism was that G9a allows DNA-damaged hepatocytes to escape p53-induced apoptosis via downregulating Bcl-G, which results in the promotion of future HCC development [52]. Moreover, Hsu et al. used liver-specific miR-122 knockout mice to demonstrate that miR-122 depletion facilitates hepatocarcinogenesis in mice receiving DEN challenge [53]. According to previous studies and our present results, we suggested that miR-122 plays the protective role in the liver from genotoxic carcinogens via targeting G9a.

In addition to the miR-122/G9a axis in HCC cells, miRNAs were also reported to be regulated by epigenetic modifications such as DNA methylation, RNA alterations, and histone modifications [26]. For example, the long non-coding (lnc)RNA, HOTAIR, was shown to suppress miR-122 expression in HCC cells via a histone methyltransferase, Enhancer of Zeste homolog 2 (EZH2)-induced upregulation of DNMTs, and further mediated DNA methylation of miR-122 [54]. G9a was reported to bind DNMT1 and regulate lung cancer stemness via maintaining DNA methylation of multiple lung cancer stem cell genes [55]. Therefore, we suggest that miR-122 might be controlled at the epigenetic level by G9a and DNMTs, and this possibility needs to be investigated in the future. In addition to miR-122, other miRNAs, such as miR-1 and miR-217, were reported to target G9a [21,56]. Moreover, our TCGA analysis also suggested that G9a may be regulated at the genomic and DNA methylation levels. Therefore, the regulatory mechanisms involved in G9a expression in HCC are intricate and encompass multiple levels.

Our clinical assessments suggested that G9a is likely to be an independent prognostic marker of HCC, albeit this result did not reach statistical significance (*p* = 0.08). This observation was supported by an independent study by Bai et al., which analyzed 350 HCC patients [20]. Their results suggested that G9a expression, stage, and the α-fetoprotein (AFP) level are independent markers of HCC. However, our data indicated that portal vein involvement is the only parameter that reached significance in our multivariate analysis. These discrepancies may have been due to the cohort size or the composition of enrolled patients. Bai’s cohort had 43% of patients in late stages, while ours had only 25% patients in late stages. The divergent values of clinical parameters may also have affected results from different analyses. For example, Qin et al. indicated that G9a expression was significantly higher in HCC patients with serum AFP of >300 ng/mL [56], while Bai et al. showed higher G9a levels in HCC patients with AFP levels of >4000 ng/mL [20]. In our results, the cutoff value of AFP was set to 500 ng/mL, and G9a expression showed a trend of being positively correlated with AFP levels (*p* = 0.09). Despite the discrepancies of the association between G9a expression levels and clinicopathological features of HCC from different studies, their results and ours all support G9a possibly serving as a potential prognostic marker of HCC.

## 5. Conclusions

Our current study confirmed the clinical importance of G9a as a potential biomarker and therapeutic target of HCC. The identified miR-122/G9a regulatory axis further established a unique and crucial role of G9a during HCC development and progression. A more comprehensive understanding of the mechanism involved in G9a expression will elucidate its clinical potential and can direct the development of precise anticancer medicines.

## Figures and Tables

**Table 1:** Clinicopathologic characteristics of hepatocellular carcinoma patients with low and high expression of G9a.

Parameter	G9a Low	G9a High	*p*-Value
			Chi-Squared
Age (years)	60.3 ± 12.7	62.2 ± 11.6	
Sex, no. of patients			
Male	66	80	0.3303
Female	14	10	
Tumor size			
<5 cm	53	52	0.3288
>5 cm	27	38	
Stage			
I or II	61	65	0.6723
III or IV	19	25	
Portal vein involvement			
No	72	79	0.8296
Yes	8	11	
α-Fetoprotein (ng/mL)			
0–500	67	66	0.0916
>500	11	23	
Microvessel invasion			
No	31	29	0.4665
Yes	49	61	
Cirrhosis			
No	51	42	0.0507
Yes	28	45	
Hepatitis B virus status			
Negative	25	30	0.9449
Positive	54	60	
Hepatitis C virus status			
Negative	61	66	0.6860
Positive	18	24	

**Table 2:** Univariate and multivariate analyses of potential prognostic variables.

Parameter	Comparison	Univariate Analysis		Multivariate Analysis	
		HR (95% CI)	*p*-Value	HR (95% CI)	*p*-Value
G9a score	low (≥240); high (<240)	1.52 (1.01–2.27)	0.0442 *	1.45 (0.96–2.20)	0.0805
AJCC stage	I or II; III or IV	1.71 (1.11–2.63)	0.0148 *	1.50 (0.83–2.72)	0.184
Sex	male; female	0.87 (0.49–1.55)	0.6314	1.10 (0.59–2.05)	0.7714
AFP level (ng/mL)	≥500; <500	1.56 (0.97–2.51)	0.0662	1.45 (0.85–2.49)	0.1762
Tumor size (cm)	≥5; <5	1.48 (0.99–2.20)	0.0546	1.12 (0.64–1.97)	0.6843
Portal vein involvement	yes; no	1.74 (1.15–2.63)	0.0096 *	1.81 (1.13–2.90)	0.0147 *
Microvessel invasion	yes; no	0.88 (0.59–1.33)	0.5579	0.64 (0.40–1.03)	0.0659
HBV	positive; negative	1.29 (0.86–1.95)	0.2237	1.32 (0.82–2.15)	0.2589
HCV	positive; negative	0.89 (0.57–1.41)	0.6348	1.15 (0.67–1.97)	0.6102

HR, hazard ratio; CI, confidence interval; AFP, α-fetoprotein; HBV, hepatitis B virus; HCV, hepatitis C virus. * *p* < 0.05.

## Data Availability

All data used during the current study are available from the corresponding author upon reasonable request.

## Supplementary Materials

The following are available online at https://www.mdpi.com/article/10.3390/cancers13102376/s1, Figure S1: Uncropped blots used in main figures, Table S1: Primer and probe sequences used for quantitative PCR, Table S2: The correlation between CpG methylation and G9a gene expression.

## References

[1] Simard E.P., Ward E.M., Siegel R., Jemal A. (2012). Cancers with increasing incidence trends in the United States: 1999 through 2008. CA A Cancer J. Clin..

[2] Kulik L., El-Serag H.B. (2019). Epidemiology and Management of Hepatocellular Carcinoma. Gastroenterolgy.

[3] Bray F., Ferlay J., Soerjomataram I., Siegel R.L., Torre L.A., Jemal A. (2018). Global cancer statistics 2018: GLOBOCAN estimates of incidence and mortality worldwide for 36 cancers in 185 countries. CA Cancer J. Clin..

[4] Nishida N., Goel A. (2011). Genetic and epigenetic signatures in human hepatocellular carcinoma: A systematic review. Curr. Genom..

[5] Nault J.-C., Zucman-Rossi J. (2011). Genetics of Hepatobiliary Carcinogenesis. Semin. Liver Dis..

[6] Khan F.S., Ali I., Afridi U.K., Ishtiaq M., Mehmood R. (2017). Epigenetic mechanisms regulating the development of hepatocellular carcinoma and their promise for therapeutics. Hepatol. Int..

[7] Herceg Z., Paliwal A. (2011). Epigenetic mechanisms in hepatocellular carcinoma: How environmental factors influence the epigenome. Mutat. Res./Rev. Mutat. Res..

[8] Farazi P.A., Depinho R.A. (2006). Hepatocellular carcinoma pathogenesis: From genes to environment. Nat. Rev. Cancer.

[9] Nakamura M., Chiba T., Kanayama K., Kanzaki H., Saito T., Kusakabe Y., Kato N. (2019). Epigenetic dysregulation in hepatocellular carcinoma: An up-to-date review. Hepatol. Res..

[10] Toh T.B., Lim J.J., Chow E.K. (2019). Epigenetics of hepatocellular carcinoma. Clin. Transl. Med..

[11] Tachibana M., Sugimoto K., Fukushima T., Shinkai Y. (2001). SET Domain-containing Protein, G9a, Is a Novel Lysine-preferring Mammalian Histone Methyltransferase with Hyperactivity and Specific Selectivity to Lysines 9 and 27 of Histone H3. J. Biol. Chem..

[12] Chen M.-W., Hua K.-T., Kao H.-J., Chi C.-C., Wei L.-H., Johansson G., Shiah S.-G., Chen P.-S., Jeng Y.-M., Cheng T.-Y. (2010). H3K9 Histone Methyltransferase G9a Promotes Lung Cancer Invasion and Metastasis by Silencing the Cell Adhesion Molecule Ep-CAM. Cancer Res..

[13] Hua K.-T., Wang M.-Y., Chen M.-W., Wei L.-H., Chen C.-K., Ko C.-H., Jeng Y.-M., Sung P.-L., Jan Y.-H., Hsiao M. (2014). The H3K9 methyltransferase G9a is a marker of aggressive ovarian cancer that promotes peritoneal metastasis. Mol. Cancer.

[14] Hsiao S.-M., Chen M.-W., Chen C.-A., Chien M.-H., Hua K.-T., Hsiao M., Kuo M.-L., Wei L.-H. (2015). The H3K9 Methyltransferase G9a Represses E-cadherin and is Associated with Myometrial Invasion in Endometrial Cancer. Ann. Surg. Oncol..

[15] Liu C.-W., Hua K.-T., Li K.-C., Kao H.-F., Hong R.-L., Ko J.-Y., Hsiao M., Kuo M.-L., Tan C.-T. (2017). Histone Methyltransferase G9a Drives Chemotherapy Resistance by Regulating the Glutamate–Cysteine Ligase Catalytic Subunit in Head and Neck Squamous Cell Carcinoma. Mol. Cancer Ther..

[16] Wu H., Zhang H., Wang P., Mao Z., Feng L., Wang Y., Liu C., Xia Q., Li B., Zhao H. (2013). Short-Form CDYLb but not long-form CDYLa functions cooperatively with histone methyltransferase G9a in hepatocellular carcinomas. Genes Chromosomes Cancer.

[17] Kondo Y., Shen L., Suzuki S., Kurokawa T., Masuko K., Tanaka Y., Kato H., Mizuno Y., Yokoe M., Sugauchi F. (2007). Alterations of DNA methylation and histone modifications contribute to gene silencing in hepatocellular carcinomas. Hepatol. Res..

[18] Hung S.-Y., Lin H.-H., Yeh K.-T., Chang J.-G. (2014). Histone-modifying genes as biomarkers in hepatocellular carcinoma. Int. J. Clin. Exp. Pathol..

[19] Yokoyama M., Chiba T., Zen Y., Oshima M., Kusakabe Y., Noguchi Y., Yuki K., Koide S., Tara S., Saraya A. (2017). Histone lysine methyltransferase G9a is a novel epigenetic target for the treatment of hepatocellular carcinoma. Oncotarget.

[20] Bai K., Cao Y., Huang C., Chen J., Zhang X., Jiang Y. (2016). Association of Histone Methyltransferase G9a and Overall Survival After Liver Resection of Patients With Hepatocellular Carcinoma With a Median Observation of 40 Months. Medicine.

[21] Wei L., Chiu D.K.-C., Tsang F.H.-C., Law C.-T., Cheng C.L.-H., Au S.L.-K., Lee J.M.-F., Wong C.C.-L., Ng I.O.-L., Wong C.-M. (2017). Histone methyltransferase G9a promotes liver cancer development by epigenetic silencing of tumor suppressor gene RARRES3. J. Hepatol..

[22] Hu Y., Zheng Y., Dai M., Wang X., Wu J., Yu B., Zhang H., Cui Y., Kong W., Wu H. (2019). G9a and histone deacetylases are crucial for Snail2-mediated E-cadherin repression and metastasis in hepatocellular carcinoma. Cancer Sci..

[23] Gu M., Toh T.B., Hooi L., Lim J.J., Zhang X., Chow E.K.-H. (2019). Nanodiamond-Mediated Delivery of a G9a Inhibitor for Hepatocellular Carcinoma Therapy. ACS Appl. Mater. Interfaces.

[24] Bárcena-Varela M., Caruso S., Llerena S., Álvarez-Sola G., Uriarte I., Latasa M.U., Urtasun R., Rebouissou S., Alvarez L., Jimenez M. (2019). Dual Targeting of Histone Methyltransferase G9a and DNA-Methyltransferase 1 for the Treatment of Experimental Hepatocellular Carcinoma. Hepatology.

[25] Iorio M.V., Piovan C., Croce C.M. (2010). Interplay between microRNAs and the epigenetic machinery: An intricate network. Biochim. Biophys. Acta (BBA)-Bioenerg..

[26] Arif K.M.T., Elliott E.K., Haupt L.M., Griffiths L.R. (2020). Regulatory Mechanisms of Epigenetic miRNA Relationships in Human Cancer and Potential as Therapeutic Targets. Cancers.

[27] Rebouissou S., Zucman-Rossi J., Moreau R., Qiu Z., Hui L. (2017). Note of caution: Contaminations of hepatocellular cell lines. J. Hepatol..

[28] Heffelfinger S.C., Hawkins H.H., Barrish J., Taylor L., Darlington G.J. (1992). SK HEP-1: A human cell line of endothelial origin. Vitr. Cell. Dev. Biol. Anim..

[29] López-Terrada D., Cheung S.W., Finegold M.J., Knowles B.B. (2009). Hep G2 is a hepatoblastoma-derived cell line. Hum. Pathol..

[30] Oh S.Y., Seok J.Y., Choi Y.S., Lee S.H., Bae J.-S., Lee Y.M. (2015). The Histone Methyltransferase Inhibitor BIX01294 Inhibits HIF-1α Stability and Angiogenesis. Mol. Cells.

[31] Chien M.-H., Lee W.-J., Yang Y.-C., Tan P., Pan K.-F., Liu Y.-C., Tsai H.-C., Hsu C.-H., Wen Y.-C., Hsiao M. (2018). N-α-acetyltransferase 10 protein promotes metastasis by stabilizing matrix metalloproteinase-2 protein in human osteosarcomas. Cancer Lett..

[32] Tay N., Chan S.-H., Ren E.-C. (1990). Detection of integrated hepatitis B virus DNA in hepatocellular carcinoma cell lines by nonradioactive in situ hybridization. J. Med. Virol..

[33] Chen J.-Y., Harrison T.J., Tsuei D.-J., Hsu T.-Y., Zuckerman A.J., Chan T.-S., Yang C.-S. (1994). Analysis of Integrated Hepatitis B Virus DNA and Flanking Cellular Sequences in the Hepatocellular Carcinoma Cell Line HCC36. Intervirology.

[34] Sobolewski C., Calo N.V., Portius D., Foti M. (2015). MicroRNAs in Fatty Liver Disease. Semin. Liver Dis..

[35] Satishchandran A., Szabo G. (2015). MicroRNAs in Alcoholic Liver Disease. Semin. Liver Dis..

[36] Otsuka M., Kishikawa T., Yoshikawa T., Yamagami M., Ohno M., Takata A., Shibata C., Ishibashi R., Koike K. (2016). MicroRNAs and liver disease. J. Hum. Genet..

[37] Chen Z., Xie H., Hu M., Huang T., Hu Y., Sang N., Zhao Y. (2020). Recent progress in treatment of hepatocellular carcinoma. Am. J. Cancer Res..

[38] Bayo J., Fiore E.J., Dominguez L.M., Real A., Malvicini M., Rizzo M., Atorrasagasti C., García M.G., Argemi J., Martinez E.D. (2019). A comprehensive study of epigenetic alterations in hepatocellular carcinoma identifies potential therapeutic targets. J. Hepatol..

[39] Schulze K., Imbeaud S., Letouzé E., Alexandrov L.B., Calderaro J., Rebouissou S., Couchy G., Meiller C., Shinde J., Soysouvanh F. (2015). Exome sequencing of hepatocellular carcinomas identifies new mutational signatures and potential therapeutic targets. Nat. Genet..

[40] Wen B., Wu H., Shinkai Y.A., Irizarry R., Feinberg A.P. (2009). Large histone H3 lysine 9 dimethylated chromatin blocks distinguish differentiated from embryonic stem cells. Nat. Genet..

[41] Lu H., Lei X., Zhang Q. (2019). Liver-specific knockout of histone methyltransferase G9a impairs liver maturation and dysregulates inflammatory, cytoprotective, and drug-processing genes. Xenobiotica.

[42] Zhang Y., Xue W., Zhang W., Yuan Y., Zhu X., Wang Q., Wei Y., Yang D., Yang C., Chen Y. (2020). Histone methyltransferase G9a protects against acute liver injury through GSTP1. Cell Death Differ..

[43] Zhang W., Yang D., Yuan Y., Liu C., Chen H., Zhang Y., Wang Q., Petersen R.B., Huang K., Zheng L. (2020). Muscular G9a Regulates Muscle-Liver-Fat Axis by Musclin Under Overnutrition in Female Mice. Diabetes.

[44] Barcena-Varela M., Paish H., Alvarez L., Uriarte I., Latasa M.U., Santamaria E., Recalde M., Garate M., Claveria A., Colyn L. (2020). Epigenetic mechanisms and metabolic reprogramming in fibrogenesis: Dual targeting of G9a and DNMT1 for the inhibition of liver fibrosis. Gut.

[45] Zeisel M.B., Pfeffer S., Baumert T.F. (2013). miR-122 acts as a tumor suppressor in hepatocarcinogenesis in vivo. J. Hepatol..

[46] Bandiera S., Pfeffer S., Baumert T.F., Zeisel M.B. (2015). miR-122–A key factor and therapeutic target in liver disease. J. Hepatol..

[47] Girard M., Jacquemin E., Munnich A., Lyonnet S., Henrion-Caude A. (2008). miR-122, a paradigm for the role of microRNAs in the liver. J. Hepatol..

[48] Liu A.M., Xu Z., Shek F.H., Wong K.-F., Lee N.P., Poon R.T., Chen J., Luk J.M. (2014). miR-122 Targets Pyruvate Kinase M2 and Affects Metabolism of Hepatocellular Carcinoma. PLoS ONE.

[49] Lei Y., Chen L., Zhang G., Shan A., Ye C., Liang B., Deng L. (2020). MicroRNAs target the Wnt/β-catenin signaling pathway to regulate epithelial-mesenchymal transition in cancer (Review). Oncol. Rep..

[50] Bai S., Nasser M.W., Wang B., Hsu S.-H., Datta J., Kutay H., Yadav A., Nuovo G., Kumar P., Ghoshal K. (2009). MicroRNA-122 Inhibits Tumorigenic Properties of Hepatocellular Carcinoma Cells and Sensitizes These Cells to Sorafenib. J. Biol. Chem..

[51] Nakatsuka T., Tateishi K., Kato H., Fujiwara H., Yamamoto K., Kudo Y., Nakagawa H., Tanaka Y., Ijichi H., Ikenoue T. (2021). Inhibition of histone methyltransferase G9a attenuates liver cancer initiation by sensitizing DNA-damaged hepatocytes to p53-induced apoptosis. Cell Death Dis..

[52] Hsu S.-H., Wang B., Kutay H., Bid H., Shreve J., Zhang X., Costinean S., Bratasz A., Houghton P., Ghoshal K. (2013). Hepatic Loss of miR-122 Predisposes Mice to Hepatobiliary Cyst and Hepatocellular Carcinoma upon Diethylnitrosamine Exposure. Am. J. Pathol..

[53] Cheng D., Deng J., Zhang B., He X., Meng Z., Li G., Ye H., Zheng S., Wei L., Deng X. (2018). LncRNA HOTAIR epigenetically suppresses miR-122 expression in hepatocellular carcinoma via DNA methylation. EBioMedicine.

[54] Pangeni R.P., Yang L., Zhang K., Wang J., Li W., Guo C., Yun X., Sun T., Wang J., Raz D.J. (2020). G9a regulates tumorigenicity and stemness through genome-wide DNA methylation reprogramming in non-small cell lung cancer. Clin. Epigenet..

[55] Thienpont B., Aronsen J.M., Robinson E.L., Okkenhaug H., Loche E., Ferrini A., Brien P., Alkass K., Tomasso A., Agrawal A. (2017). The H3K9 dimethyltransferases EHMT1/2 protect against pathological cardiac hypertrophy. J. Clin. Investig..

[56] Qin J., Li Q., Zeng Z., Wu P., Jiang Y., Luo T., Ji X., Zhang Q., Hao Y., Chen L. (2018). Increased expression of G9A contributes to carcinogenesis and indicates poor prognosis in hepatocellular carcinoma. Oncol. Lett..

