# Supplementary material for: Histone Methyltransferase G9a-Promoted Progression of Hepatocellular Carcinoma Is Targeted by Liver-Specific Hsa-miR-122
Source: Cancers (Basel). 2021 May 14;13(10):2376. doi: 10.3390/cancers13102376 (PMC8157135; doi:10.3390/cancers13102376)
Supplement: Supplementary file 1 [file cancers-13-02376-s001.zip › cancers-1183233-supplementary.pdf]

cancers

*Supplementary*

# Histone Methyltransferase G9a-Promoted Progression of Hepatocellular Carcinoma is Targeted by Liver-Specific Has-miR-122

**Lan-Ting Yuan et al.**

**Citation:** Yuan, L.-T.; Lee, W.-J.; Yang, Y.-C.; Chen, B.-R.; Yang, C.-Y.; Chen, M.-W.; Chen, J.-Q.; Hsiao, M.; Chien, M.-H.; Hua, K.-T.; et al. Histone Methyltransferase G9a-Promoted Progression of Hepatocellular Carcinoma Is Targeted by Liver-Specific Hsa-miR-122. *Cancers* **2021**, *13*, 2376. https://doi.org/10.3390/cancers13102376

Academic Editor: Takahiro Kodama

Received: 30 March 2021

Accepted: 11 May 2021

Published: 14 May 2021

**Publisher's Note:** MDPI stays neutral with regard to jurisdictional claims in published maps and institutional affiliations.

**Copyright:** © 2021 by the authors. Licensee MDPI, Basel, Switzerland. This article is an open access article distributed under the terms and conditions of the Creative Commons Attribution (CC BY) license (http://creativecommons.org/licenses/by/4.0/).

**Fig. 2A**

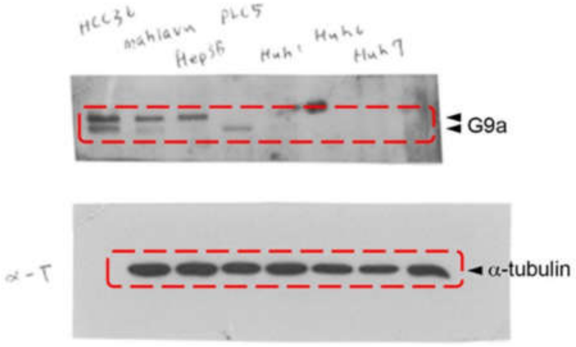


**Fig. 2B**

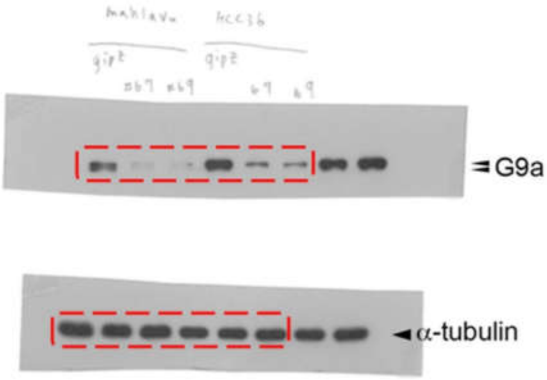


**Fig. 5C**

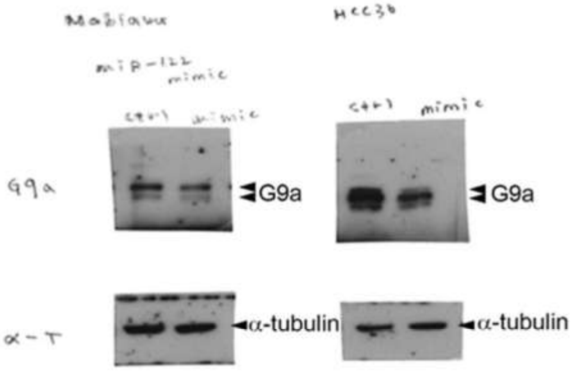


**Fig. 5D**

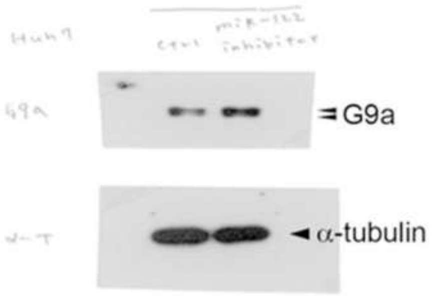


**Fig. 5F**

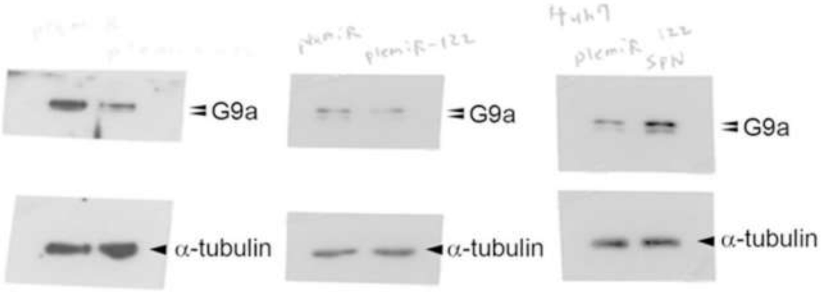


**Figure S1.** Uncropped blots used in main figures.

**Table 1.** Primer and probe sequences used for quantitative PCR.

| Gene | Primer sequences (5'-3') |
|---|---|
| *G9a/EHMT2* | (F) GGAGCCACGAGGGGTGTCCA |
| | (R) CGGCATTGCAGCCTGACAGC |
| *ACTIN* | (F) GGCGGCACCACCATGTACCCT |
| | (R) AGGGGCCGGACTCGTCATACT |
| **Gene** | **Probe sequences (5'-3')** |
| *has-miR-122* | UGGAGUGUGACAAUGGUGUUUG (AB Assay ID 002245) |
| *RUN6B* | CGCAAGGATGACACGCAAATTCGTG AAGCGTTCCATATTTTT (AB Assay ID 001093) |

**Table S2.** The correlation between CpG methylation and G9a gene expression.

| CpG sites* | Spearman's coefficient of rank correlation (rho) (95%CI) | Significance |
|---|---|---|
| cg07829740 | −0.356 (−0.442 to −0.264) | $p < 0.0001$ |
| cg07498938 | −0.297 (−0.387 to −0.201) | $p < 0.0001$ |
| cg21190306 | −0.29 (−0.380 to −0.194) | $p < 0.0001$ |
| cg02758274 | −0.282 (−0.373 to −0.185) | $p < 0.0001$ |
| cg08848598 | −0.28 (−0.371 to −0.183) | $p < 0.0001$ |
| cg21087321 | −0.276 (−0.367 to −0.179) | $p < 0.0001$ |
| cg07898120 | −0.273 (−0.364 to −0.176) | $p < 0.0001$ |
| cg17756143 | −0.271 (−0.362 to −0.174) | $p < 0.0001$ |
| cg03146965 | −0.262 (−0.355 to −0.165) | $p < 0.0001$ |
| cg27363691 | −0.261 (−0.353 to −0.163) | $p < 0.0001$ |
| cg22240884 | −0.259 (−0.352 to −0.162) | $p < 0.0001$ |
| cg04637007 | −0.258 (−0.351 to −0.160) | $p < 0.0001$ |
| cg21831287 | −0.255 (−0.347 to −0.157) | $p < 0.0001$ |
| cg03164052 | −0.254 (−0.346 to −0.156) | $p < 0.0001$ |
| cg03945301 | −0.253 (−0.346 to −0.155) | $p < 0.0001$ |
| cg19770715 | −0.252 (−0.345 to −0.154) | $p < 0.0001$ |
| cg21356750 | −0.251 (−0.344 to −0.153) | $p < 0.0001$ |

* The CpG sites within G9a promoter region and gene locus with rho <−0.25 and $p < 0.0001$ are shown.
